# Serum uric acid and the risk of major adverse cardiovascular events and death among older adults: a population-based prospective cohort study

**DOI:** 10.1186/s12877-025-06479-3

**Published:** 2025-10-15

**Authors:** Antonios Douros, Muhammad Helmi Barghouth, Damiano Ferrari, Natalie Ebert, Nina Mielke, Elke Schaeffner

**Affiliations:** 1 Institute of Clinical Pharmacology and Toxicology, Charité - Universitätsmedizin Berlin, Berlin, Germany; 2 Department of Epidemiology, Biostatistics and Occupational Health, McGill University, Montreal, QC Canada; 3 School of Epidemiology and Public Health, University of Ottawa, Ottawa, OΝ Canada; 4 Institute of Public Health, Charité - Universitätsmedizin Berlin, Berlin, Germany

**Keywords:** Geriatric nephrology, Geriatric epidemiology, Myocardial infarction, Stroke

## Abstract

**Background:**

The relationship between serum uric acid (SUA) and adverse outcomes in advanced age remains poorly understood. Our population-based prospective cohort study assessed the potential association between SUA levels and the risk of major adverse cardiovascular events (MACE) and all-cause mortality among community-dwelling older adults.

**Methods:**

We used data from the Berlin Initiative Study linked to administrative claims and vital statistics. Cohort members were followed from cohort entry (2009) until the occurrence of a study outcome or the end of the study period (2021). We created three exposure groups according to the baseline SUA distribution (in mg/dL; lower: 1.68–5.16, intermediate: 5.17–6.83, higher: 6.84-13.0); SUA levels were updated biennially. Time-dependent Cox models yielded hazard ratios (HRs) and 95% confidence intervals (CIs) of MACE and all-cause mortality adjusted for potential confounders. Sensitivity analyses addressed time-dependent confounding.

**Results:**

Our cohort included 2,058 individuals (mean age 80 years, 53% female). Lower vs. intermediate SUA levels were not associated with the risk of MACE (HR, 1.16; 95% CI, 0.88–1.54) or all-cause mortality (HR, 1.06; 95% CI, 0.86–1.31). Higher vs. intermediate SUA levels were not associated with the risk of MACE (HR, 1.11; 95% CI, 0.85–1.45) but with an increased risk of all-cause mortality (HR, 1.26; 95% CI, 1.03–1.53). Sensitivity analyses showed no statistically significant associations between higher vs. intermediate SUA levels and the risk of mortality (HR [95% CI]: 1.09 [0.89–1.34] & 1.07 [0.86–1.34]).

**Conclusion:**

Lower or higher SUA levels are not associated with the risk of MACE or all-cause mortality in older adults.

**Supplementary Information:**

The online version contains supplementary material available at 10.1186/s12877-025-06479-3.

## Introduction

Uric acid is generated within cells as the end product of purine metabolism. Subsequently, it enters the circulation and exists as serum uric acid (SUA) [1]. To date, several preclinical studies have indicated that SUA may play an important role in the development of arterial hypertension via a wide range of mechanisms including the activation of the renin-angiotensin-aldosterone system, oxidative stress, and loss of endothelial nitric oxide [1]. As a result, SUA has attracted increasing attention as a potential risk factor of cardiovascular disease.

SUA levels have been reported to increase with age [2–4]. Recent population-based data from Austria showed that the prevalence of hyperuricemia among older adults may rise up to 25% among male and 15% among female individuals [3]. Hence, there is a need to better understand the potential association between elevated SUA levels and the risk of adverse cardiovascular outcomes in this age group. To date, several observational studies have been conducted in this regard, with their findings consistently supporting an association [5–7]. Indeed, higher SUA levels have been associated with an up to 39% increase in the risk of cardiovascular death and an up to 32% increase in the risk of all-cause mortality [5–7]. Interestingly, the relationship between SUA and adverse cardiovascular outcomes seemed to be U-shaped, with lower levels also showing consistent associations with increased risks (up to 52% for cardiovascular death and up to 46% for all-cause mortality) [5–7]. 

However, these observational studies had several methodological limitations such as important exposure misclassification and considerable confounding [5–7]. Thus, the interpretation of their findings is challenging. Moreover, their clinical and public health implications remain unclear, especially with respect to whether asymptomatic variations of SUA levels constitute a risk factor of cardiovascular disease among older adults. To address this important knowledge gap, we conducted a population-based study assessing the potential association between SUA levels and the risk of major adverse cardiovascular events (MACE) and all-cause mortality in a well-characterized prospective cohort of community-dwelling older adults.

## Materials and methods

### Study source and population

We used data from the Berlin Initiative Study (BIS), a population-based cohort study that was initiated in 2009 to prospectively assess chronic kidney disease (CKD) among 2,069 community-dwelling older adults. Inclusion criteria of the BIS were membership in the statutory health insurance fund “AOK Nordost – Die Gesundheitskasse” and age of at least 70 years [8]. Exclusion criteria of the BIS were requirement for nursing care or kidney replacement therapy in the form of dialysis or transplantation at baseline. The study was approved by the Ethics Committee at Charité – Universitätsmedizin Berlin (EA2/009/08) and was conducted in alignment with the Helsinki Declaration on Medical Research Involving Human Subjects.

For the analyses presented here, in addition to the aforementioned inclusion and exclusion criteria of the BIS, we also required individuals to have a valid assessment of SUA at baseline and linkage to individual-level healthcare claims data. Participants were followed up from the date of their inclusion in the BIS (‘baseline’) until the date of the outcome, two years after their last attended BIS visit, four years after their last visit with measurement of SUA levels (see below), or the end of study period (2021), whichever occurred first.

### Exposure definition

SUA was measured via the standardized enzymatic colorimetric method (UA plus cobas^®^ assay) using a Roche/Hitachi modular analyzer (Roche Deutschland Holding GmbH, Baden-Württemberg, Germany). Based on the distribution of SUA among the total study population at baseline, we created tertiles of SUA levels and operationalized them as an ordinal categorical variable with the following groups: tertile 1 with SUA values 1.68–5.16 mg/dl (‘lower SUA levels’); tertile 2 with SUA values 5.17–6.48 mg/dl (‘intermediate SUA levels’); and tertile 3 with SUA values 6.49-13.00 mg/dl (‘higher SUA levels’). Given the population-based character of the BIS, the distribution of SUA levels in our study should approximate the distribution of SUA levels in the older German general population. We used a time-varying exposure definition, where participants were allowed to contribute person-time to > 1 exposure groups over time. SUA values were updated at every biennial visit during follow-up. Missing SUA values were imputed using the ‘last observation carried forward’ approach. For participants with consecutive missing SUA values (*n* = 33; Table S1), only the first missing value was imputed using the observed value from the preceding visit. A detailed illustration of the approach we used for missing SUA values during follow-up is shown in Figure S1.

### Outcome definition

The two study outcomes were MACE and all-cause mortality. MACE was defined as a composite endpoint consisting of non-fatal stroke, non-fatal myocardial infarction (MI), and cardiovascular death. For non-fatal stroke and non-fatal MI, we used individual-level healthcare claims data and self-reported information by the participants. Self-reported information was validated based on official hospital reports. Non-fatal stroke was defined using the 10th Revision of the International Classification of Diseases, German Modification (ICD-10-GM) codes I61, I63, and I64; non-fatal MI was defined using the ICD-10 GM codes I21 and I22. For cardiovascular and all-cause mortality, we used individual-level healthcare claims data, which were validated based on death certificates (available in 88% of the cases). We also used the confidential part of death certificates to determine the cause of death. A cardiovascular cause of death was defined as death due to MI, coronary heart disease, cerebrovascular disease or peripheral vascular disease and was assessed by two independent physicians (NE and ES); any disagreements were resolved through discussion.

### Covariate assessment

In the BIS, covariates were assessed based on primary data complemented by secondary data (i.e., individual-level healthcare claims data from the insurance fund). Primary data included self-reported information by BIS participants on sociodemographic variables, lifestyle variables, and comorbidities that were collected at baseline using a standardized questionnaire. They also included measurements of anthropometric variables, clinically relevant biomarkers, and geriatric assessments.

For the current analysis, we selected potential confounders based on subject matter expertise and literature search. We included the following sociodemographic and anthropometric variables: age (modeled flexibly via B splines), sex, level of general and vocational education (low, intermediate, high) according to the Comparative Analysis of Social Mobility in Industrial Nations scale [9], and body mass index (continuous variable). We also included the following lifestyle variables: history of smoking (binary variable), history of alcohol intake (binary variable), and frequency of physical activity (less than once per week, 1–2 times per week, 3–5 times per week, or more than 5 times per week). In addition, we included two renal parameters: estimated glomerular filtration rate (eGFR) using the BIS2 equation (modeled flexibly via B splines) [10] as a measure of kidney function and urine albumin to creatinine ratio (ACR) as a measure of kidney damage. Moreover, we included the following comorbidities: treated arterial hypertension, diabetes mellitus, hyperlipidemia, active cancer, prior MI, prior stroke, heart failure, and peripheral vascular disease. Covariate definitions are shown in Table S2. Finally, we assessed use of SUA related medications at baseline (allopurinol, febuxostat, probenecid, benzbromarone) and reported it in a descriptive fashion. Given the lack of an association between these medications and the risk of the study outcomes, we did not deem them to be confounders and, therefore, did not include them in the statistical models.

### Statistical analysis

We stratified baseline characteristics according to SUA tertiles. Categorical variables were presented as absolute and relative frequencies; continuous variables were presented as mean with standard deviation or median with interquartile range according to their distribution. We used Cox proportional hazards regression with time-fixed covariates and SUA tertiles as a time-varying exposure to estimate crude and confounder-adjusted hazard ratios (HRs) along with the corresponding 95% confidence intervals (95% CIs) of the study outcomes. We compared lower versus intermediate SUA levels and higher versus intermediate SUA levels, with intermediate SUA levels serving as reference group. Person-time was also used to calculate incidence rates for the different exposure groups. The proportional hazards assumption was assessed using the Schoenfeld residuals test [11]. 

### Secondary analyses

We conducted three secondary analyses to assess potential effect measure modifications. First, we stratified by diabetes mellitus status at baseline given the complex interplay between SUA levels and insulin resistance and the higher prevalence of hyperuricemia among patients with type 2 diabetes compared to non-diabetic populations [12]. Second, we stratified by age (70-<80 years, ≥ 80 years) given that SUA levels tend to increase over the course of life [2–4]. Third, we stratified by sex given the differences between men and women regarding the incidence of hyperuricemia related conditions such as gout [13]. 

### Sensitivity analyses

We conducted two pre-specified sensitivity analyses to account for the potential impact of time-dependent confounding. Time-dependent confounding is a bias that occurs after cohort entry and can be augmented when applying time-varying exposure definitions. First, we used Cox proportional hazards regression with time-fixed covariates and SUA tertiles as a time-varying exposure and additionally adjusting for two renal parameters reflecting kidney function and kidney damage (eGFR, ACR) modelled as time-varying covariates. Second, because time-updated eGFR and ACR may lie in the causal pathway between exposure (SUA levels) and study outcomes (MACE, all-cause mortality) and thus be mediators and not ‘true’ confounders [1, 14], we used marginal structural Cox proportional hazards models with eGFR and ACR as time-varying covariates and inverse probability weighting [15]. Extreme weights were trimmed at 10, which resulted in the truncation of < 0.2% for stabilized weights.

We also conducted two post-hoc sensitivity analyses. First, we redefined our exposure based on the reference range (2.6-6.0 mg/dl for females; 3.5–7.2 mg/dl for males [16]) used in routine clinical practice (lower, within, or higher than the reference range). Second, we used directed acyclic graphs for covariate selection.

### Supplementary analysis

We conducted a supplementary analysis, where SUA levels were modeled flexibly using B splines to account for potential non-linear associations with the study outcomes. All statistical analyses were conducted using R (Version 4.3.1; R Foundation for Statistical Computing, Vienna, Austria). The study was reported according to the Strengthening the Reporting of Observational Studies in Epidemiology (STROBE) statement (Table S3).

## Results

**Fig. 1:**
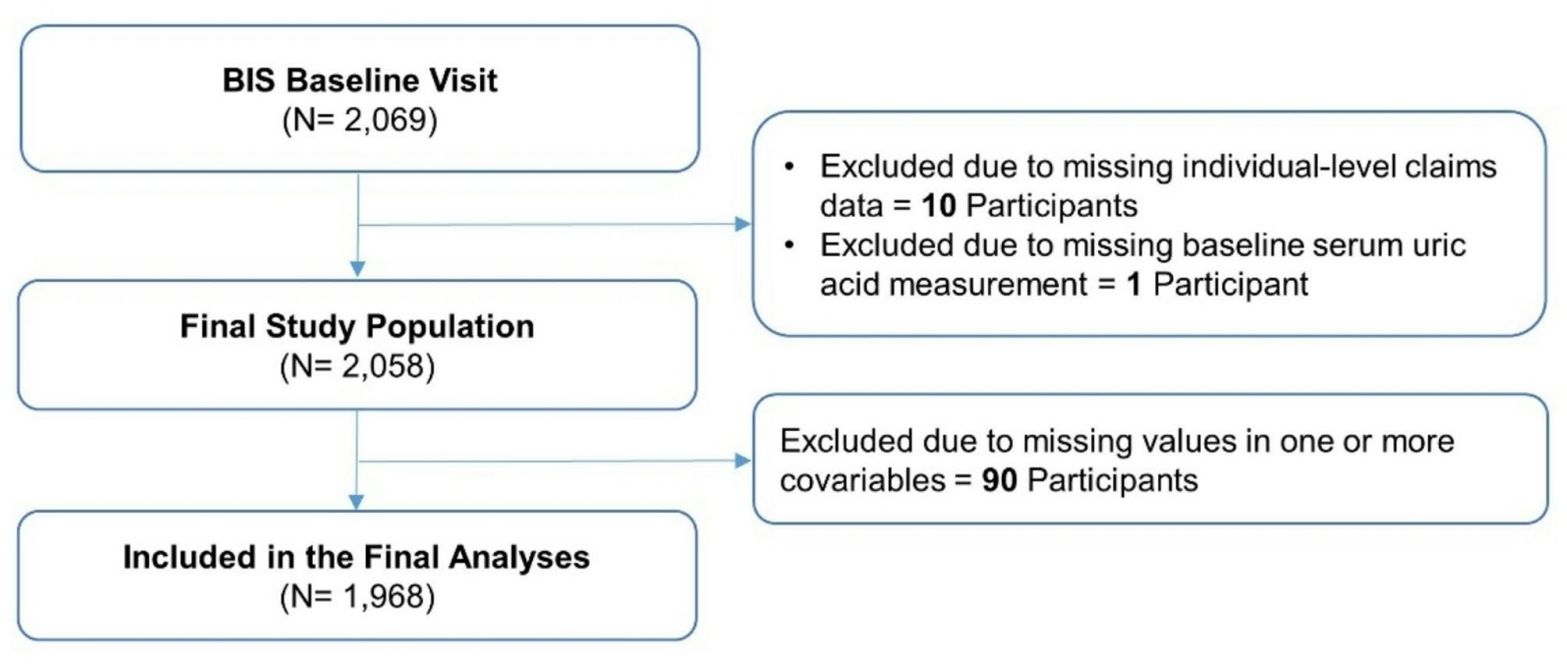
Flowchart showing the construction of the study cohort

Overall, the study cohort included 2,058 participants of the BIS (Fig. 1). Study cohort members had a mean (standard deviation) age of 80.4 (6.7) years, and 1,083 (53%) of them were female (Table 1). The mean (standard deviation) eGFR of 58.1 (15.2) mL/min per 1.73m [2] and median (interquartile range) ACR of 10.8 (4.5, 30.6) mg/g reflected their advanced age. Study cohort members with higher SUA levels at baseline were more likely to be male, to be obese, to have ever smoked, to exercise less frequently, to have decreased kidney function, and to have been diagnosed with cardiovascular disease or diabetes mellitus. However, they were less likely to have been diagnosed with hyperlipidemia. There were no differences in the use of SUA related medications between different exposure groups, with allopurinol being the only relevant medication reported (in 10–12% of participants depending on the exposure group).

**Table 1:** Baseline characteristics stratified by SUA levels at cohort entry

Characteristic^a^	Total *N* = 2058	SUA tertile 1 (1.68–5.16 mg/dl) *N* = 686	SUA tertile 2 (5.17–6.83 mg/dl) *N* = 689	SUA tertile 3 (6.84-13.0 mg/dl) *N* = 683
Demographics				
Age in years, mean (SD)	80.4 (6.7)	79.7 (6.7)	80.2 (6.6)	81.3 (6.7)
Female sex	1083 (53%)	482 (70%)	353 (51%)	248 (36%)
Education level^b^				
Low	1242 (60%)	401 (58%)	429 (62%)	412 (60%)
Intermediate	405 (20%)	159 (23%)	122 (18%)	124 (18%)
High	402 (20%)	121 (18%)	137 (20%)	144 (21%)
Missing	9 (0.4%)	5 (0.7%)	1 (0.1%)	3 (0.4%)
Lifestyle variables				
Body mass index in kg/m^2^				
< 30	1513 (74%)	542 (79%)	501 (73%)	470 (69%)
≥ 30	544 (26%)	143 (21%)	188 (27%)	213 (31%)
Missing	1 (0.0%)	1 (0.1%)	0 (0%)	0 (0%)
Smoking status				
Never	1032 (50%)	416 (61%)	338 (49%)	278 (41%)
Ever	1024 (50%)	269 (39%)	350 (51%)	405 (59%)
Missing	2 (0.1%)	1 (0.1%)	1 (0.1%)	0 (0%)
Alcohol consumption				
Yes	1527 (74%)	504 (73%)	503 (73%)	520 (76%)
Missing	12 (0.6%)	6 (0.9%)	4 (0.6%)	2 (0.3%)
Frequency of physical activity				
Less than once per week	525 (26%)	145 (21%)	189 (27%)	191 (28%)
1–2 times per week	430 (21%)	129 (19%)	143 (21%)	158 (23%)
3–5 times per week	526 (26%)	173 (25%)	175 (25%)	178 (26%)
More than 5 times per week	572 (28%)	237 (35%)	179 (26%)	156 (23%)
Missing	5 (0.2%)	2 (0.3%)	3 (0.4%)	0 (0%)
Markers of kidney function				
eGFR_BIS2_ in mL/min per 1.73m^2^, mean (SD)	58.1 (15.2)	65.4 (13.3)	58.3 (13.4)	50.5 (15.2)
ACR in mg/g, median (IQR)	10.8 (4.5, 30.6)	9.6 (4.7, 24.7)	9.9 (4.3, 28.0)	13.5 (4.5, 41.7)
Comorbidities				
Treated arterial hypertension	1626 (79%)	470 (69%)	544 (79%)	612 (90%)
Missing	7 (0.3%)	3 (0.4%)	2 (0.3%)	2 (0.3%)
Prior myocardial infarction	476 (23%)	111 (16%)	169 (25%)	196 (29%)
Missing	21 (1.0%)	5 (0.7%)	6 (0.9%)	10 (1.5%)
Prior stroke	277 (13%)	77 (11%)	83 (12%)	117 (17%)
Missing	22 (1.1%)	9 (1.3%)	9 (1.3%)	4 (0.6%)
Peripheral artery disease	451 (22%)	121 (18%)	146 (21%)	184 (27%)
Missing	17 (0.8%)	6 (0.9%)	6 (0.9%)	5 (0.7%)
Heart failure	1243 (60%)	332 (48%)	424 (62%)	487 (71%)
Diabetes mellitus	538 (26%)	150 (22%)	172 (25%)	216 (32%)
Missing	3 (0.1%)	1 (0.1%)	1 (0.1%)	1 (0.1%)
Hyperlipidemia	579 (28%)	227 (33%)	189 (27%)	163 (24%)
Active cancer	385 (19%)	117 (17%)	133 (19%)	135 (20%)
SUA related medications^c^				
Allopurinol	222 (11%)	66 (10%)	80 (12%)	76 (11%)

*Abbreviations*: *SUA* Serum uric acid, *SD* Standard deviation, *IQR* Interquartile range, *ACR* Albumin-to-creatine ratio, *CASMIN* Comparative analysis of social mobility in industrial nations; eGFR, estimated glomerular filtration rate, *BIS* Berlin Initiative Study

^a^Values are numbers (%) unless stated otherwise. Missing values for any of the covariates did not exceed 1% in any of the exposure groups

^b^Education level was defined based on the CASMIN scale

^c^SUA related medications are reported descriptively but were not included in the statistical analyses; none of the participants in our study population reported using febuxostat, probenecid, or benzbromarone

Median (interquartile range) duration of follow-up in the study cohort was 6.4 (3.2, 10.1) for MACE and 7.9 (4.0, 10.1) for all-cause mortality. Table 2 shows that when compared to intermediate SUA levels, lower SUA levels were not associated with the risk of MACE (crude incidence rates per 100 person-years: 2.33 versus 2.46; adjusted HR, 1.16; 95% CI, 0.88–1.54) or with the risk of all-cause mortality (crude incidence rates per 100 person-years: 3.63 versus 3.97; adjusted HR, 1.06; 95% CI, 0.86–1.31). When compared to intermediate SUA levels, higher SUA levels were not associated with the risk of MACE (crude incidence rates per 100 person-years: 3.29 versus 2.46; adjusted HR, 1.11; 95% CI, 0.85–1.45) but were associated with an increased risk of all-cause mortality (crude incidence rates per 100 person-years: 6.88 versus 3.97; adjusted HR, 1.26; 95% CI, 1.03–1.53). The results of the Schoenfeld residuals test suggested that the proportional hazards assumption was likely met (global p-values: 0.74 for MACE, 0.05 for all-cause mortality).

**Table 2:** Risk of the study outcomes associated with SUA levels among community-dwelling older adults (primary analysis)

SUA tertiles^a^	*N* Events	*N* Person-years	Incidence rate (per 100 person-years)	Crude HR (95% CI)	Adjusted^b^ HR (95% CI)
MACE					
SUA tertile 1 (lower levels)	102	4376	2.33	0.95 (0.72–1.24)	1.16 (0.88–1.54)
SUA tertile 2 (intermediate levels)	102	4153	2.46	Reference	Reference
SUA tertile 3 (higher levels)	135	4104	3.29	1.35 (1.05–1.74)	1.11 (0.85–1.45)
All-cause mortality					
SUA tertile 1 (lower levels)	170	4681	3.63	0.91 (0.73–1.12)	1.06 (0.86–1.31)
SUA tertile 2 (intermediate levels)	177	4464	3.97	Reference	Reference
SUA tertile 3 (higher levels)	309	4491	6.88	1.76 (1.46–2.11)	1.26 (1.03–1.53)

*Abbreviations*: *SUA* Serum uric acid, *MACE* Major adverse cardiovascular events, *HR* Hazard ratio, *CI* Confidence interval, *eGFR* estimated glomerular filtration rate, *BIS* Berlin Initiative Study

^a^The thresholds for the SUA tertiles were based on the distribution of SUA in the total population at baseline; tertile 1: 1.68–5.16 mg/dl, tertile 2: 5.17–6.83 mg/dl, and tertile 3: 6.84-13.0 mg/dl

^b^Adjusted for the following time-fixed covariates: age, sex, education level, body mass index, smoking, alcohol consumption, physical activity, eGFR_BIS2_, albumin-to-creatinine ratio, treated arterial hypertension, prior myocardial infarction, prior stroke, peripheral artery disease, heart failure, diabetes mellitus, hyperlipidemia, and active cancer

In secondary analyses, stratification by age showed no major effect measure modification for MACE; however, the increase in the risk of all-cause mortality associated with higher versus intermediate SUA levels was higher among participants aged 70-<80 years (adjusted HR, 1.69; 95% CI, 1.18–2.42) than those aged ≥ 80 years (adjusted HR, 1.20; 95% CI, 0.96–1.51) (p-value for interaction: 0.04) (Table S4). Stratification by diabetes or sex did not show any major effect measure modifications for either study outcome (Table 3, Table S5).

**Table 3:** Risk of the study outcomes associated with SUA levels among community-dwelling older adults (stratified by diabetes mellitus status)

SUA tertiles^a^	*N* Events	*N* Person-years	Incidence rate (per 100 person-years)	Crude HR (95% CI)	Adjusted^b^ HR (95% CI)	*P*-values for interaction
Diabetes mellitus						
MACE						
SUA tertile 1 (lower levels)	22	825	2.67	0.94 (0.54–1.65)	1.09 (0.62–1.92)	
SUA tertile 2 (intermediate levels)	29	1011	2.87	Reference	Reference	
SUA tertile 3 (higher levels)	45	1211	3.72	1.30 (0.82–2.07)	1.16 (0.73–1.86)	
All-cause mortality						
SUA tertile 1 (lower levels)	45	879	5.12	1.01 (0.68–1.49)	0.96 (0.66–1.41)	
SUA tertile 2 (intermediate levels)	56	1092	5.13	Reference	Reference	
SUA tertile 3 (higher levels)	95	1339	7.09	1.40 (1.02–1.94)	1.14 (0.81–1.60)	
No diabetes mellitus						
MACE						
SUA tertile 1 (lower levels)	80	3551	2.25	0.96 (0.70–1.32)	1.17 (0.84–1.62)	0.73
SUA tertile 2 (intermediate levels)	73	3143	2.32	Reference	Reference	
SUA tertile 3 (higher levels)	90	2893	3.11	1.35 (0.99–1.84)	1.09 (0.79–1.52)	0.61
All-cause mortality						
SUA tertile 1 (lower levels)	125	3802	3.29	0.90 (0.70–1.16)	1.10 (0.85–1.42)	0.57
SUA tertile 2 (intermediate levels)	121	3372	3.59	Reference	Reference	
SUA tertile 3 (higher levels)	214	3151	6.79	1.90 (1.52–2.38)	1.33 (1.05–1.69)	0.38

*Abbreviations*: *SUA* Serum uric acid, *MACE* Major adverse cardiovascular events, *HR* Hazard ratio, *CI* Confidence interval, *eGFR* estimated glomerular filtration rate, *BIS* Berlin Initiative Study

^a^The thresholds for the SUA tertiles were based on the distribution of SUA in the total population at baseline; tertile 1: 1.68–5.16 mg/dl, tertile 2: 5.17–6.83 mg/dl, and tertile 3: 6.84-13.0 mg/dl

^**b**^Adjusted for the following time-fixed covariates: age, sex, education level, body mass index, smoking, alcohol consumption, physical activity, eGFR_BIS2_, albumin-to-creatinine ratio, treated arterial hypertension, prior myocardial infarction, prior stroke, peripheral artery disease, heart failure, diabetes mellitus, hyperlipidemia, and active cancer

In sensitivity analyses, both the time-dependent adjustment for eGFR and ACR (adjusted HR, 1.09; 95% CI, 0.89–1.34) and the marginal structural model analysis (adjusted HR, 1.07; 95% CI, 0.86–1.34) led to the disappearance of the increase in the risk of all-cause mortality associated with higher versus intermediate SUA levels, while other results remained largely unchanged (Table S6). The post-hoc sensitivity analyses and the supplementary analysis led to findings that were consistent with those of the primary analysis (Table S7, Figures S2-S3).

## Discussion

Our large population-based prospective cohort study showed that besides a 26% increased risk of all-cause mortality with higher SUA levels, higher or lower levels of this biomarker were not associated with the risk of adverse clinical outcomes among older adults. Moreover, the increased risk of all-cause mortality with higher SUA levels disappeared after accounting for time-dependent confounding.

The potential role of SUA in the development of adverse clinical outcomes was initially assessed among middle-aged individuals, with observational studies showing an association between higher SUA levels and increased risks of cardiovascular and all-cause mortality [17–19]. Due to the high prevalence of hyperuricemia among older adults, there has been strong interest to corroborate the findings obtained from middle-aged individuals in this age group. Indeed, several observational studies seem to support the notion that the role of SUA as a cardiovascular risk factor is retained in advanced age, with both lower and higher SUA levels being associated with increased risks of cardiovascular and all-cause mortality [5–7]. 

Of note, observational studies in the area had several methodological limitations such as important exposure misclassification and strong confounding [5–7]. Regarding misclassification of exposure, it was possibly introduced because SUA levels were only measured at baseline, with potential changes in the biomarker during follow-up not being considered in the analyses. Generally, this bias can lead to spurious associations especially in settings where the exposure is dynamic and the follow-up is long. This seems to be the case here, given that SUA levels have been shown to change even in advanced age [3], while the studies in question had relatively extended follow-up periods (up to 11 years) [5–7]. Importantly, SUA levels tend to increase rather than decrease with rising age; hence, exposure misclassification is likely to be differential between groups, which makes inferences about the directionality of the resulting bias challenging and complicates the interpretation of the respective findings.

Residual confounding due to unmeasured or inappropriately estimated confounders such as comedications, frailty, functional level, or kidney function may also have affected the validity of previous studies. For example, functional level is a well-established risk factor of adverse cardiovascular outcomes in advanced age and could also be associated with SUA levels [20], which makes it an important confounder in this setting. However, previous studies did not consider any measures of functional level in their statistical analyses [5–7]. Kidney function is another important confounder due to its association both with SUA levels and the risk of adverse cardiovascular outcomes [21–23]. The use of creatinine-based and not cystatin C-based eGFR equations in previous studies may have led to an overestimation of kidney function due to the elevated prevalence of sarcopenia among older adults [6]. 

Our results do not support prior studies regarding the role of SUA in advanced age. While we did observe a moderately increased risk of all-cause mortality associated with higher SUA levels (more pronounced among participants aged 70-<80 years), higher SUA levels were not associated with the risk of MACE, and there were also no associations between lower SUA levels and the risk of either study outcome. Moreover, the increased risk of all-cause mortality with higher SUA levels was not corroborated in sensitivity analyses controlling for time-dependent confounding.

Our results suggest that variations in SUA levels among older adults do not constitute a risk factor of cardiovascular disease or death, and that age may modify the association between SUA levels and adverse clinical outcomes, with increased risks observed among middle-aged individuals possibly being attenuated or minimized in advanced age. Of note, the increased risk of all-cause mortality among septuagenarians but not among octogenarians in our study seems to support this hypothesis. A potential explanation could be related to a phenomenon known as depletion of susceptibles [24], where higher-risk individuals, i.e., those susceptible to MACE or death due to higher or lower SUA levels, have already been depleted from older populations such as the BIS cohort. That said, we are not aware of mechanistic data in support of this notion. Finally, there was a numerical difference in the effect estimates for the association between higher SUA levels and the risk of all-cause mortality between patients with and without diabetes mellitus. However, given that the interaction analysis was not statistically significant, we think that this difference should be interpreted as a random variation across strata rather than true effect measure modification by diabetes.

Based on our results, pharmacologic treatment with the sole aim of normalizing SUA levels in the absence of clinical symptoms does not seem justified. This is in line with evidence from randomized trials showing that pharmacologic treatment of asymptomatic hyperuricemia does not lead to clinical benefits [25, 26]. It is also in line with the recent KDIGO guidelines that do not recommend the use of SUA lowering medications in people with CKD and asymptomatic hyperuricemia to delay CKD progression [27]. Overall, our results are clinically relevant given the high prevalence of hyperuricemia among older adults [2–4], the toxicities related to the use of SUA lowering medications [28], and the considerable burden of polypharmacy in advanced age.

Our study has strengths. First, the application of a time-varying exposure definition that incorporated multiple measurements of SUA during follow-up alleviated misclassification of exposure, which was a major limitation of previous studies. Second, the utilization of the population-based prospective BIS cohort as data source likely maximized the external validity of our findings. Third, the definition of our study outcomes was based on a combination of self-reported data, linked healthcare claims data, and adjudication by experts; this likely minimized misclassification of the outcome.

Our study also has potential limitations. First, residual confounding cannot be excluded in the absence of randomization. However, we went to great lengths to minimize this bias by including a wide range of potential confounders in our statistical analyses such as frequency of physical activity as a proxy of functional level and GFR estimation based on the combination of creatinine and cystatin C. Moreover, we conducted sensitivity analyses additionally controlling for time-dependent confounding. Second, we did not assess whether patients had symptoms related to hyperuricemia such as gout. However, looking at the low utilization rates of SUA lowering medications at baseline, we can assume that the majority of individuals was symptom free. Third, some of the secondary analyses did not yield precise estimates. Therefore, the respective results should be considered hypothesis generating.

## Conclusions

The totality of our study findings does not support an association between lower or higher SUA levels and the risk of MACE or all-cause mortality among older adults. Therefore, a role of SUA as a significant cardiovascular risk factor in advanced age seems unlikely. Moreover, pharmacological treatment to normalize the levels of this biomarker in the absence of clinical symptoms does not seem justified.

## Supplementary Information

Supplementary Material 1.

## Data Availability

The datasets used and analyzed during the study are available from ES on reasonable request.

## Abbreviations

SUA: serum uric acid
MACE: major adverse cardiovascular events
BIS: Berlin Initiative Study
CKD: chronic kidney disease
MI: myocardial infarction
ICD-10 GM: International Classification of Diseases, German Modification, 10^th^ Revision
eGFR: estimated glomerular filtration rate
ACR: albumin to creatinine ratio
HR: hazard ratio
CI: confidence interval
STROBE: Strengthening the Reporting of Observational Studies in Epidemiology
