# Supplementary material for: Serum uric acid and the risk of major adverse cardiovascular events and death among older adults: a population-based prospective cohort study
Source: BMC Geriatr. 2025 Oct 15;25:778. doi: 10.1186/s12877-025-06479-3 (PMC12523046; doi:10.1186/s12877-025-06479-3)
Supplement: Supplementary file 1 — Supplementary Material 1. [file 12877_2025_6479_MOESM1_ESM.docx]

**Table of contents**

[Figure S1. Handling of missing values and determination of person-time 2](#_Toc207039857)

[Figure S2. Risk of the study outcomes associated with serum uric acid levels (modeled as a continuous variable via B-splines) 3](#_Toc207039858)

[Figure S3. Directed acyclic graph illustrating the causal framework underlying the association between serum uric acid levels and the risk of all-cause mortality 4](#_Toc207039859)

[Table S1. Overview of the missing values for serum uric acid among study participants 5](#_Toc207039860)

[Table S2. Definition of covariates 6](#_Toc207039861)

[Table S3. STROBE statement 7](#_Toc207039862)

[Table S4. Risk of the outcomes associated with SUA levels among community-dwelling older adults (stratification by age) 9](#_Toc207039863)

[Table S5. Risk of the outcomes associated with SUA levels among community-dwelling older adults (stratification by sex) 10](#_Toc207039864)

[Table S6. Risk of the outcomes associated with SUA levels among community-dwelling older adults (pre-specified sensitivity analyses) 11](#_Toc207039865)

[Table S7. Risk of the outcomes associated with SUA levels among community-dwelling older adults (post-hoc sensitivity analyses) 12](#_Toc207039866)

# **Figure S1. Handling of missing values and determination of person-time**

*
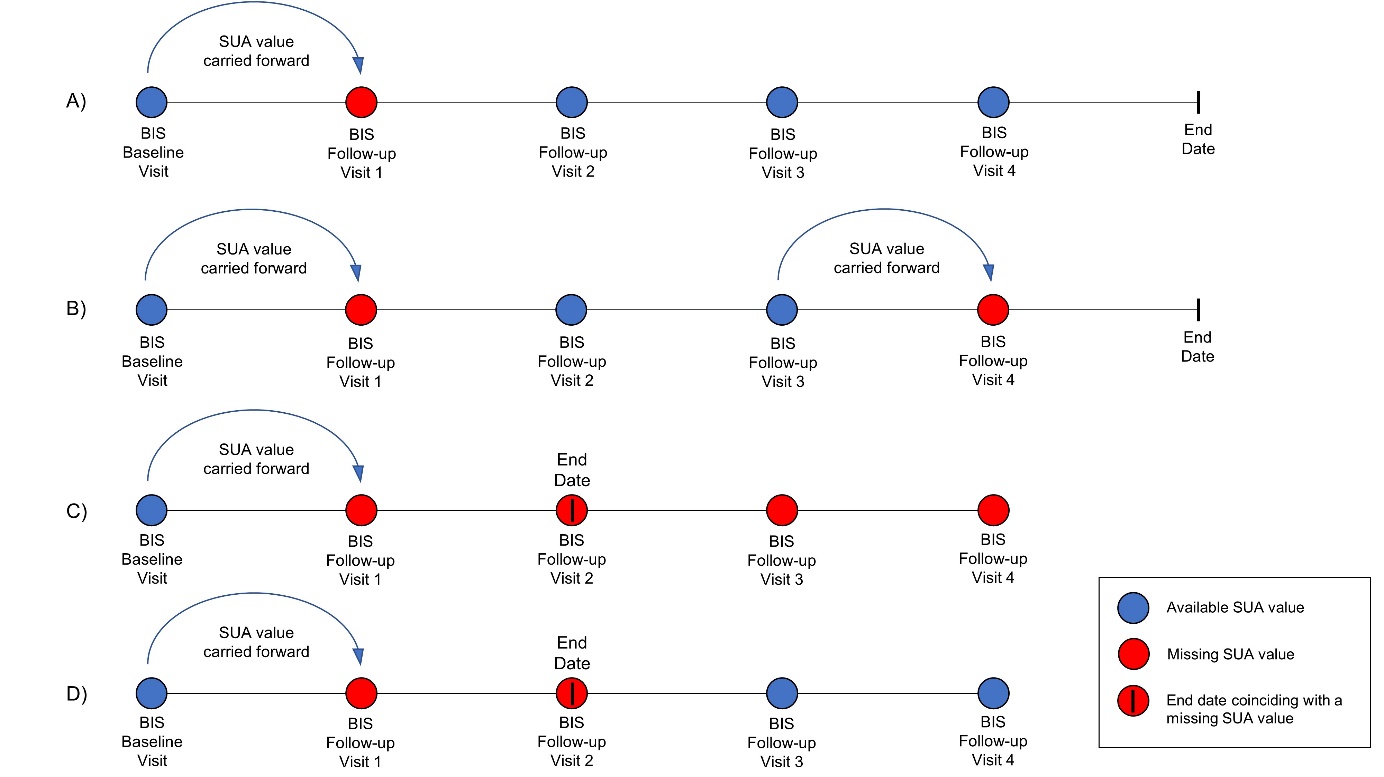
*

Abbreviations: SUA, serum uric acid; BIS, Berlin Initiative Study.

In scenarios A and B, non-consecutive SUA values were imputed based on the last observed value allowing the end date to exceed the BIS follow-up period. Whereas in scenarios C and D, due to consecutive missing SUA values, participants were censored four years after the visit whose SUA value was carried forward.

# **Figure S2. Risk of the study outcomes associated with serum uric acid levels (modeled as a continuous variable via B-splines)**

**
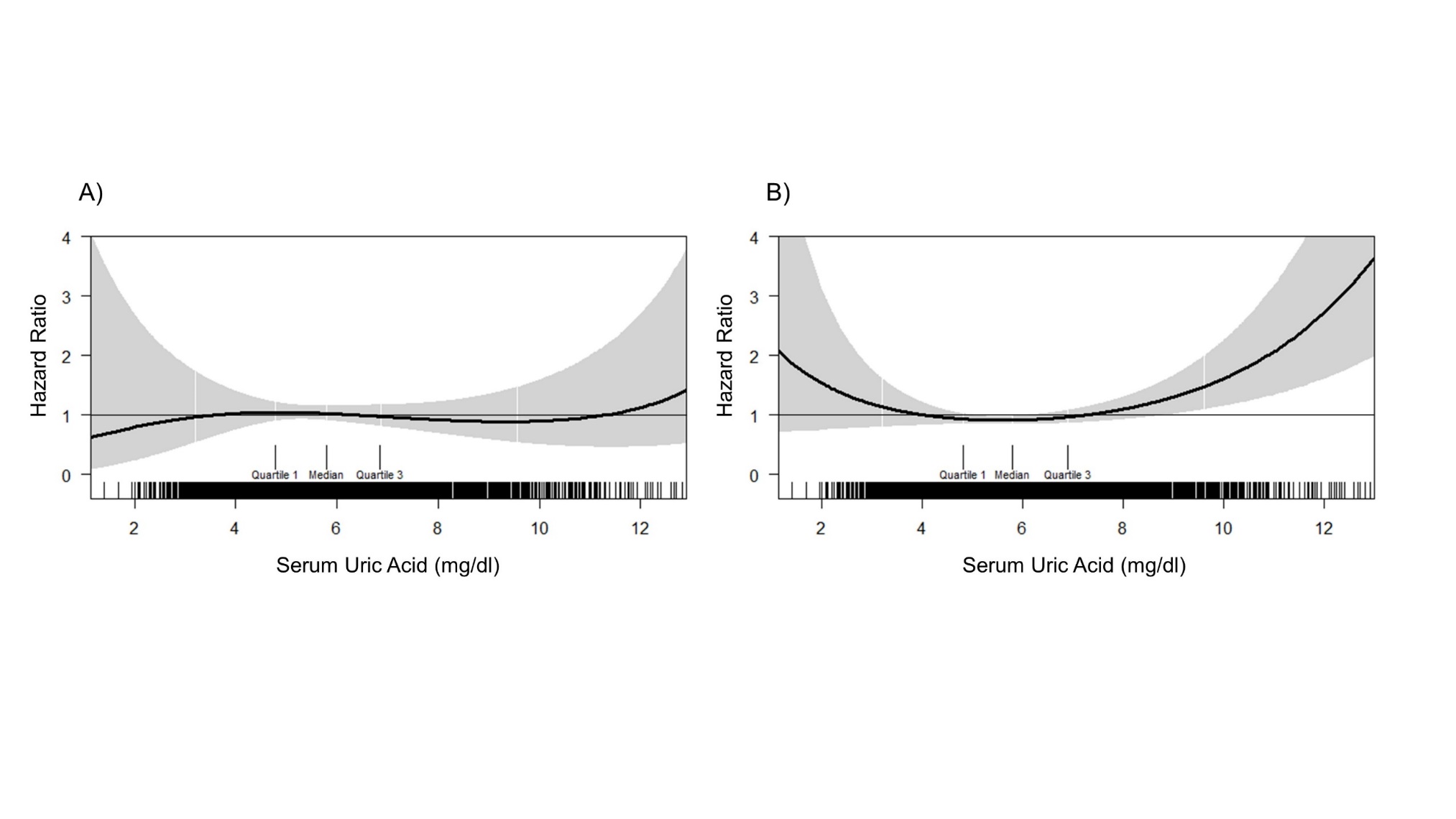
**

Abbreviations: eGFR, estimated glomerular filtration rate; BIS, Berlin Initiative Study.

Panel A: Major adverse cardiovascular events. Panel B: All-cause mortality

In both panels, the black lines represent mean adjusted hazard ratios. The light grey areas represent the corresponding 95% confidence intervals.

Adjusted for the following time-fixed covariates: age, sex, education level, body mass index, smoking, alcohol consumption, physical activity, eGFR_BIS2_, albumin-to-creatinine ratio, treated arterial hypertension, prior myocardial infarction, prior stroke, peripheral artery disease, heart failure, diabetes mellitus, hyperlipidemia, and active cancer.

# **Figure S3. Directed acyclic graph illustrating the causal framework underlying the association between serum uric acid levels and the risk of all-cause mortality**


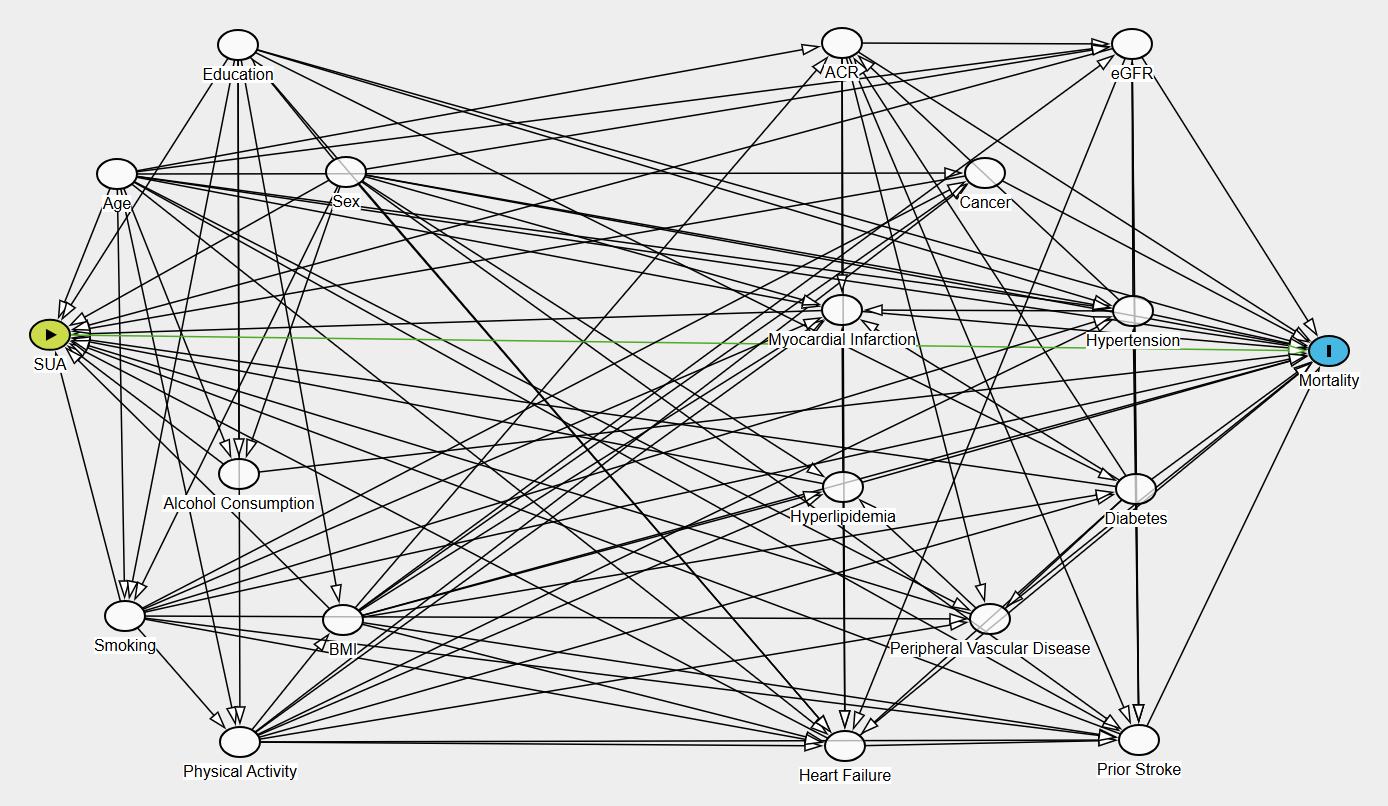


Abbreviations: eGFR, estimated glomerular filtration rate; BMI, body mass index; ACR, albumin-to-creatinine ratio; SUA, serum uric acid.

# **Table S1. Overview of the missing values for serum uric acid among study participants**

|  | Number of missing values | | | | | |
| --- | --- | --- | --- | --- | --- | --- |
|  | None | One | Two | Three | Four | Total |
| No missing values | 1887 | 0 | 0 | 0 | 0 | 1887 |
| Non-consecutive missing values | 0 | 136 | 2 | 0 | 0 | 138 |
| Two consecutive missing values | 0 | 0 | 24 | 2 | 0 | 26 |
| Three consecutive missing values | 0 | 0 | 0 | 6 | 0 | 6 |
| Four consecutive missing values | 0 | 0 | 0 | 0 | 1 | 1 |
| Total | 1887 | 136 | 26 | 8 | 1 | 2058 |

# **Table S2. Definition of covariates**

| **Covariate** | **Definition** |
| --- | --- |
| Lifestyle variables (history of smoking, history of alcohol intake, frequency of physical activity) | Self-reported data at baseline |
| Biomarkers of kidney function (estimated glomerular filtration rate, urine albumin-to-creatinine ratio) | Measurements at baseline and during follow-up |
| Treated arterial hypertension | Regular intake of any antihypertensive medication (self-reported data at baseline) |
| Diabetes mellitus | Regular intake of any antidiabetic medication (self-reported data at baseline) OR glycosylated hemoglobin ≥6.5% (measurement at baseline) |
| Hyperlipidemia | Regular intake of any lipid-lowering medication (self-reported data at baseline) OR total cholesterol ≥240 mg/dL (measurement at baseline) OR low-density lipoprotein cholesterol ≥160 mg/dL (measurement at baseline) |
| Active cancer | ICD-10 GM diagnostic code for cancer within the year preceding inclusion in the BIS excluding non-melanoma skin cancer (C44) |
| Prior myocardial infarction | ICD-10 GM diagnostic codes for myocardial infarction (I21, I22, I23, I25.2) |
| Prior stroke | ICD-10 GM diagnostic codes for stroke (I61, I63, I64, I69.1-4) |
| Heart failure | ICD-10 GM diagnostic codes for heart failure (I11.0, I13.0, I13.2, I25, I50) |
| Peripheral vascular disease | ICD-10 GM diagnostic codes for peripheral vascular disease (I70.2, I73.1, I73.9) OR prior angiography, intermittent claudication, peripheral vascular disease-induced bypass or amputation (self-reported data at baseline) |

Abbreviations: ICD-10-GM, 10^th^ Revision of the International Classification of Diseases, German Modification.

# **Table S3. STROBE statement**

|  | Item No | Recommendation |
| --- | --- | --- |
| **Title and abstract** | 1 | (*a*) Indicate the study’s design with a commonly used term in the title or the abstract ✓ |
|  |  | (*b*) Provide in the abstract an informative and balanced summary of what was done and what was found ✓ |
| Introduction | | |
| Background/rationale | 2 | Explain the scientific background and rationale for the investigation being reported ✓ |
| Objectives | 3 | State specific objectives, including any prespecified hypotheses ✓ |
| Methods | | |
| Study design | 4 | Present key elements of study design early in the paper ✓ |
| Setting | 5 | Describe the setting, locations, and relevant dates, including periods of recruitment, exposure, follow-up, and data collection ✓ |
| Participants | 6 | (*a*) *Cohort study*—Give the eligibility criteria, and the sources and methods of selection of participants. Describe methods of follow-up ✓  *Case-control study*—Give the eligibility criteria, and the sources and methods of case ascertainment and control selection. Give the rationale for the choice of cases and controls (N/A)  *Cross-sectional study*—Give the eligibility criteria, and the sources and methods of selection of participants (N/A) |
|  |  | (*b*) *Cohort study*—For matched studies, give matching criteria and number of exposed and unexposed (N/A)  *Case-control study*—For matched studies, give matching criteria and the number of controls per case (N/A) |
| Variables | 7 | Clearly define all outcomes, exposures, predictors, potential confounders, and effect modifiers. Give diagnostic criteria, if applicable ✓ |
| Data sources/ measurement | 8 | For each variable of interest, give sources of data and details of methods of assessment (measurement). Describe comparability of assessment methods if there is more than one group ✓ |
| Bias | 9 | Describe any efforts to address potential sources of bias ✓ |
| Study size | 10 | Explain how the study size was arrived at ✓ |
| Quantitative variables | 11 | Explain how quantitative variables were handled in the analyses. If applicable, describe which groupings were chosen and why ✓ |
| Statistical methods | 12 | (*a*) Describe all statistical methods, including those used to control for confounding ✓ |
|  |  | (*b*) Describe any methods used to examine subgroups and interactions ✓ |
|  |  | (*c*) Explain how missing data were addressed ✓ |
|  |  | (*d*) *Cohort study*—If applicable, explain how loss to follow-up was addressed (N/A)  *Case-control study*—If applicable, explain how matching of cases and controls was addressed (N/A)  *Cross-sectional study*—If applicable, describe analytical methods taking account of sampling strategy (N/A) |
|  |  | (*e*) Describe any sensitivity analyses ✓ |
| **Results** | | |
| Participants | 13 | (a) Report numbers of individuals at each stage of study—e.g., numbers potentially eligible, examined for eligibility, confirmed eligible, included in the study, completing follow-up, and analysed ✓ |
|  |  | (b) Give reasons for non-participation at each stage ✓ |
|  |  | (c) Consider use of a flow diagram ✓ |
| Descriptive data | 14 | (a) Give characteristics of study participants (e.g., demographic, clinical, social) and information on exposures and potential confounders ✓ |
|  |  | (b) Indicate number of participants with missing data for each variable of interest ✓ |
|  |  | (c) *Cohort study*—Summarise follow-up time (e.g., average and total amount) ✓ |
| Outcome data | 15 | *Cohort study*—Report numbers of outcome events or summary measures over time ✓ |
|  |  | *Case-control study—*Report numbers in each exposure category, or summary measures of exposure (N/A) |
|  |  | *Cross-sectional study—*Report numbers of outcome events or summary measures (N/A) |
| Main results | 16 | (*a*) Give unadjusted estimates and, if applicable, confounder-adjusted estimates and their precision (e.g., 95% confidence interval). Make clear which confounders were adjusted for and why they were included ✓ |
|  |  | (*b*) Report category boundaries when continuous variables were categorized ✓ |
|  |  | (*c*) If relevant, consider translating estimates of relative risk into absolute risk for a meaningful time period (N/A) |
| Other analyses | 17 | Report other analyses done— e.g., analyses of subgroups and interactions, and sensitivity analyses ✓ |
| **Discussion** | | |
| Key results | 18 | Summarise key results with reference to study objectives ✓ |
| Limitations | 19 | Discuss limitations of the study, taking into account sources of potential bias or imprecision. Discuss both direction and magnitude of any potential bias ✓ |
| Interpretation | 20 | Give a cautious overall interpretation of results considering objectives, limitations, multiplicity of analyses, results from similar studies, and other relevant evidence ✓ |
| Generalisability | 21 | Discuss the generalisability (external validity) of the study results ✓ |
| **Other information** | | |
| Funding | 22 | Give the source of funding and the role of the funders for the present study and, if applicable, for the original study on which the present article is based ✓ |

# **Table S4. Risk of the outcomes associated with SUA levels among community-dwelling older adults (stratification by age)**

| **SUA tertiles^*^** | **N Events** | **N**  **Person-years** | **Incidence rate**  **(per 100 person-years)** | **Crude HR**  **(95% CI)** | **Adjusted^**^ HR**  **(95% CI)** | **P-values for interaction** |
| --- | --- | --- | --- | --- | --- | --- |
| **Age 70-<80 years** |  |  |  |  |  |  |
| **MACE** |  |  |  |  |  |  |
| SUA tertile 1 | 43 | 2815 | 1.53 | 0.76 (0.51-1.14) | 0.92 (0.60-1.42) |  |
| SUA tertile 2 | 50 | 2499 | 2.00 | Reference | Reference |  |
| SUA tertile 3 | 59 | 2169 | 2.72 | 1.40 (0.96-2.04) | 1.19 (0.81-1.74) |  |
| **All-cause mortality** |  |  |  |  |  |  |
| SUA tertile 1 | 50 | 2983 | 1.68 | 0.92 (0.62-1.36) | 1.14 (0.76-1.72) |  |
| SUA tertile 2 | 48 | 2684 | 1.79 | Reference | Reference |  |
| SUA tertile 3 | 83 | 2343 | 3.54 | 2.04 (1.43-2.90) | 1.69 (1.18-2.42) |  |
| **Age ≥80 years** |  |  |  |  |  |  |
| **MACE** |  |  |  |  |  |  |
| SUA tertile 1 | 59 | 1561 | 3.78 | 1.20 (0.83-1.73) | 1.27 (0.86-1.87) | 0.22 |
| SUA tertile 2 | 52 | 1654 | 3.14 | Reference | Reference |  |
| SUA tertile 3 | 76 | 1935 | 3.93 | 1.24 (0.88-1.76) | 1.18 (0.82-1.70) | 0.80 |
| **All-cause mortality** |  |  |  |  |  |  |
| SUA tertile 1 | 120 | 1699 | 7.06 | 0.97 (0.76-1.25) | 0.98 (0.77-1.26) | 0.80 |
| SUA tertile 2 | 129 | 1780 | 7.26 | Reference | Reference |  |
| SUA tertile 3 | 226 | 2147 | 10.53 | 1.45 (1.17-1.80) | 1.20 (0.96-1.51) | 0.04 |

Abbreviations: SUA, serum uric acid; MACE, major adverse cardiovascular events; HR, hazard ratio; CI, confidence interval; eGFR, estimated glomerular filtration rate; BIS, Berlin Initiative Study.

^*^ The thresholds for the SUA tertiles were based on the distribution of SUA in the total population at baseline; tertile 1: 1.68-5.16 mg/dl, tertile 2: >5.16-6.83 mg/dl, and tertile 3: 6.83-13.0 mg/dl.

^**^ Adjusted for the following time-fixed covariates: age, sex, education level, body mass index, smoking, alcohol consumption, physical activity, eGFR_BIS2_, albumin-to-creatinine ratio, treated arterial hypertension, prior myocardial infarction, prior stroke, peripheral artery disease, heart failure, diabetes mellitus, hyperlipidemia, and active cancer.

# **Table S5. Risk of the outcomes associated with SUA levels among community-dwelling older adults (stratification by sex)**

| **SUA tertiles^*^** | **N Events** | **N**  **Person-years** | **Incidence rate**  **(per 100 person-years)** | **Crude HR**  **(95% CI)** | **Adjusted^**^ HR**  **(95% CI)** | **P-values for interaction** |
| --- | --- | --- | --- | --- | --- | --- |
| **Female sex** |  |  |  |  |  |  |
| **MACE** |  |  |  |  |  |  |
| SUA tertile 1 | 63 | 3155.951 | 2.00 | 1.10 (0.74-1.63) | 1.42 (0.94-2.17) |  |
| SUA tertile 2 | 41 | 2250.979 | 1.82 | Reference | Reference |  |
| SUA tertile 3 | 47 | 1650.229 | 2.85 | 1.55 (1.02-2.36) | 1.26 (0.80-1.98) |  |
| **All-cause mortality** |  |  |  |  |  |  |
| SUA tertile 1 | 87 | 3336.862 | 2.61 | 0.82 (0.60-1.11) | 1.06 (0.76-1.46) |  |
| SUA tertile 2 | 76 | 2390.032 | 3.18 | Reference | Reference |  |
| SUA tertile 3 | 96 | 1797.150 | 5.34 | 1.68 (1.24-2.28) | 1.48 (1.07-2.05) |  |
| **Male sex** |  |  |  |  |  |  |
| **MACE** |  |  |  |  |  |  |
| SUA tertile 1 | 39 | 1219.881 | 3.20 | 0.98 (0.66-1.47) | 1.05 (0.70-1.56) | 0.35 |
| SUA tertile 2 | 61 | 1902.246 | 3.21 | Reference | Reference |  |
| SUA tertile 3 | 88 | 2453.473 | 3.59 | 1.14 (0.82-1.58) | 1.03 (0.74-1.43) | 0.18 |
| **All-cause mortality** |  |  |  |  |  |  |
| SUA tertile 1 | 83 | 1344.470 | 6.17 | 1.24 (0.93-1.66) | 1.14 (0.86-1.52) | 0.78 |
| SUA tertile 2 | 101 | 2073.893 | 4.87 | Reference | Reference |  |
| SUA tertile 3 | 213 | 2693.489 | 7.91 | 1.65 (1.30-2.08) | 1.23 (0.96-1.57) | 0.60 |

Abbreviations: SUA, serum uric acid; MACE, major adverse cardiovascular events; HR, hazard ratio; CI, confidence interval; eGFR, estimated glomerular filtration rate; BIS, Berlin Initiative Study.

^*^ The thresholds for the SUA tertiles were based on the distribution of SUA in the total population at baseline; tertile 1: 1.68-5.16 mg/dl, tertile 2: >5.16-6.83 mg/dl, and tertile 3: 6.83-13.0 mg/dl.

^**^ Adjusted for the following time-fixed covariates: age, sex, education level, body mass index, smoking, alcohol consumption, physical activity, eGFR_BIS2_, albumin-to-creatinine ratio, treated arterial hypertension, prior myocardial infarction, prior stroke, peripheral artery disease, heart failure, diabetes mellitus, hyperlipidemia, and active cancer.

# **Table S6. Risk of the outcomes associated with SUA levels among community-dwelling older adults (pre-specified sensitivity analyses)**

| **SUA tertiles^*^** | **N**  **Events** | **N**  **Person-years** | **Incidence rate**  **(per 100 person-years)** | **Crude HR**  **(95% CI)** | **Adjusted HR**  **(95% CI)** |
| --- | --- | --- | --- | --- | --- |
| **Time-dependent adjustment^**^** |  |  |  |  |  |
| **MACE** |  |  |  |  |  |
| SUA tertile 1 | 102 | 4376 | 2.33 | 0.95 (0.72-1.24) | 1.12 (0.84-1.49) |
| SUA tertile 2 | 102 | 4153 | 2.46 | Reference | Reference |
| SUA tertile 3 | 135 | 4104 | 3.29 | 1.35 (1.05-1.74) | 1.05 (0.80-1.39) |
| **All-cause mortality** |  |  |  |  |  |
| SUA tertile 1 | 170 | 4681 | 3.63 | 0.84 (0.68-1.04) | 1.05 (0.84-1.32) |
| SUA tertile 2 | 177 | 4464 | 3.97 | Reference | Reference |
| SUA tertile 3 | 309 | 4491 | 6.88 | 1.55 (1.29-1.86) | 1.09 (0.89-1.34) |
| **Marginal structural model** |  |  |  |  |  |
| **MACE** |  |  |  |  |  |
| SUA tertile 1 | 102 | 4376 | 2.33 | 0.95 (0.72-1.24) | 1.09 (0.78-1.51) |
| SUA tertile 2 | 102 | 4153 | 2.46 | Reference | Reference |
| SUA tertile 3 | 135 | 4104 | 3.29 | 1.35 (1.05-1.74) | 1.02 (0.75-1.38) |
| **All-cause mortality** |  |  |  |  |  |
| SUA tertile 1 | 170 | 4681 | 3.63 | 0.84 (0.68-1.04) | 1.02 (0.76-1.36) |
| SUA tertile 2 | 177 | 4464 | 3.97 | Reference | Reference |
| SUA tertile 3 | 309 | 4491 | 6.88 | 1.55 (1.29-1.86) | 1.07 (0.86-1.34) |

Abbreviations: SUA, serum uric acid; MACE, major adverse cardiovascular events; HR, hazard ratio; CI, confidence interval; eGFR, estimated glomerular filtration rate; BIS, Berlin Initiative Study.

^*^ The thresholds for the SUA tertiles were based on the distribution of SUA in the total population at baseline; tertile 1: 1.68-5.16 mg/dl, tertile 2: >5.16-6.83 mg/dl, and tertile 3: 6.83-13.0 mg/dl.

^**^ Adjusted for the following time-fixed covariates: age, sex, education level, body mass index, smoking, alcohol consumption, physical activity, eGFR_BIS2_, albumin-to-creatinine ratio, treated arterial hypertension, prior myocardial infarction, prior stroke, peripheral artery disease, heart failure, diabetes mellitus, hyperlipidemia, and active cancer. Also adjusted for eGFR_BIS2_ and for albumin-to-creatinine ratio as time-dependent covariates.

# **Table S7. Risk of the outcomes associated with SUA levels among community-dwelling older adults (post-hoc sensitivity analyses)**

| **SUA categories** | **N**  **Events** | **N**  **Person-years** | **Incidence rate**  **(per 100 person-years)** | **Crude HR**  **(95% CI)** | **Adjusted^**^ HR**  **(95% CI)** |
| --- | --- | --- | --- | --- | --- |
| **Redefined exposure^*^** |  |  |  |  |  |
| **MACE** |  |  |  |  |  |
| SUA (lower than reference range) | 5 | 140 | 3.57 | 1.42 (0.56-3.59) | 1.43 (0.55-3.75) |
| SUA (within reference Range) | 218 | 8741 | 2.49 | Reference | Reference |
| SUA (higher than reference range) | 116 | 3752 | 3.09 | 1.25 (1.00-1.57) | 1.04 (0.81-1.33) |
| **All-cause mortality** |  |  |  |  |  |
| SUA (lower than reference range) | 13 | 164 | 7.93 | 1.84 (1.06-3.19) | 1.49 (0.83-2.66) |
| SUA (within reference Range) | 380 | 9369 | 4.06 | Reference | Reference |
| SUA (higher than reference range) | 263 | 4103 | 6.41 | 1.60 (1.36-1.87) | 1.17 (0.98-1.39) |
| **Minimal adjustment set** |  |  |  |  |  |
| **MACE** |  |  |  |  |  |
| SUA tertile 1 | 102 | 4376 | 2.33 | 0.95 (0.72-1.24) | 1.12 (0.84-1.49) |
| SUA tertile 2 | 102 | 4153 | 2.46 | Reference | Reference |
| SUA tertile 3 | 135 | 4104 | 3.29 | 1.35 (1.05-1.74) | 1.12 (0.86-1.46) |
| **All-cause mortality** |  |  |  |  |  |
| SUA tertile 1 | 170 | 4681 | 3.63 | 0.91 (0.73-1.12) | 1.09 (0.88-1.34) |
| SUA tertile 2 | 177 | 4464 | 3.97 | Reference | Reference |
| SUA tertile 3 | 309 | 4491 | 6.88 | 1.76 (1.46-2.11) | 1.25 (1.03-1.51) |

Abbreviations: SUA, serum uric acid; MACE, major adverse cardiovascular events; HR, hazard ratio; CI, confidence interval; eGFR, estimated glomerular filtration rate; BIS, Berlin Initiative Study.

^*^ The thresholds for the SUA categories were based on reference ranges used in routine clinical practice (2.6-6.0 mg/dL for females; 3.5-7.2 mg/dL for males).

^**^ Adjusted for the following time-fixed covariates: age, sex, education level, body mass index, smoking, alcohol consumption, physical activity, eGFR_BIS2_, albumin-to-creatinine ratio, treated arterial hypertension, prior myocardial infarction, prior stroke, peripheral artery disease, heart failure, diabetes mellitus, hyperlipidemia, and active cancer.
